# Supplementary material for: Failure mechanism of monolayer graphene under hypervelocity impact of spherical projectile
Source: Sci Rep. 2016 Sep 13;6:33139. doi: 10.1038/srep33139 (PMC5020609; doi:10.1038/srep33139)
Supplement: Supplementary Information [file srep33139-s1.doc]

**Supporting Information**

**Failure mechanism of monolayer graphene under hypervelocity impact of spherical projectile**

Kang Xia^1^, Haifei Zhan*^,1^, De’an Hu^2^, and Yuantong Gu^**,1^

^1^School of Chemistry, Physics and Mechanical Engineering, Queensland University of Technology (QUT), Brisbane QLD 4001, Australia

^2^State Key Laboratory of Advanced Design and Manufacturing for Vehicle Body, Hunan University, Changsha 410082, PR China

**S1. The time step influence**

Different time steps ranging from 0.05 to 1 fs under an impact velocity of 6 and 3km/s, have been examined. As shown in Figure 1, the relative penetration energy fluctuates within 5%, and no clear relationship is found between the relative penetration energy and the examined time step. By assessing the atomic configurations, similar deformation pattern and number of breaking bonds are observed. In all, the simulation results are independent on the selected time step.


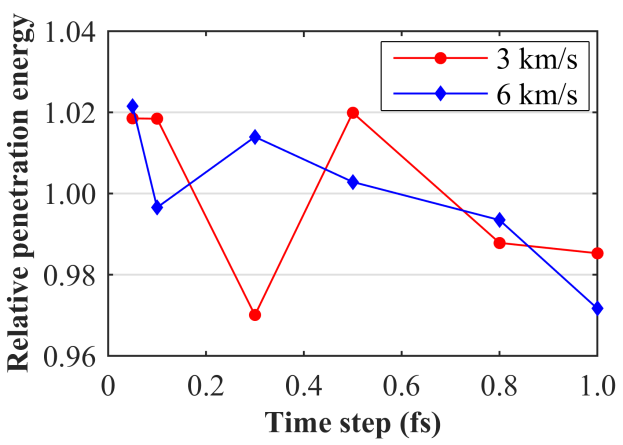


**Figure 1** Estimated relative penetration energy under different time steps ranging from 0.05 to 1 fs. The relative penetration energy is estimated from $E_{pr}=E_{p}/E_{pa}$, here *E_pa_* represents the average penetration energy for all examined time steps.

**S2. Fixed-boundary conditions and sample size**

Figure 2a compares the relative specific penetration among all examined cases, which fluctuates around one (within ± 10%). More importantly, no clear relation is shown between the relative specific penetration and the impact velocity while changing the fixed-boundary condition. In addition, the fluctuation of the specific penetration also contains the influence from the sample size of the graphene membrane as the considered graphene sizes are not exactly the same under different fixed-boundary conditions. From Figure 2b, the graphene size leads to marginal influence (within ± 5%) to the relative penetration energy when the side length increases from 20 to 60 nm (for the square sample). These results have suggested that although the influence from different fixed-boundary condition on the estimated penetration energy (at different velocities) vary from each other, the influence is insignificant.


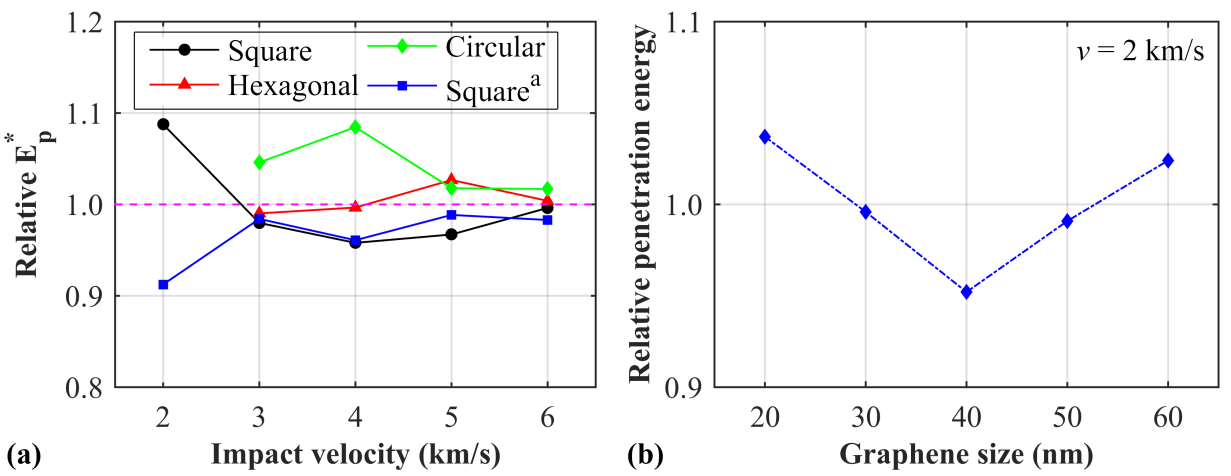


**Figure 2** (a) The relative specific penetration energy $E_{pr}^{*}$ as a function of impact velocity while altering the fixed-boundary conditions. Here, the relative specific penetration energy is estimated from $E_{pr}^{*}=E_{p}^{*}/E_{pa}^{*}$, where $E_{pa}^{*}$ is the average of the specific penetration energy under different fixed-boundary conditions (with the same impact velocity). (b) The relative penetration energy as estimated from the graphene sample size ranging from 20 to 60 nm (under the impact velocity of 2 km/s).
